# Supplementary material for: NMR Metabolite Profiling for the Characterization of Vessalico Garlic Ecotype and Bioactivity against Xanthomonas campestris pv. campestris
Source: Plants (Basel). 2024 Apr 23;13(9):1170. doi: 10.3390/plants13091170 (PMC11085173; doi:10.3390/plants13091170)
Supplement: Supplementary file 1 [file plants-13-01170-s001.zip › plants-2938432-supplementary.pdf]

NMR metabolite profiling for a fast characterization of Vessalico garlic ecotype and bioactivity against *Xanthomonas campestris* pv. *campestris*

Valeria Iobbi[1], Valentina Parisi[2], Anna Paola Lanteri[3], Norbert Maggi[4], Mauro Giacomini[4], Giuliana Drava[1], Giovanni Minuto[3], Andrea Minuto[3], Nunziatina De Tommasi[2], Angela Bisio[1]

[1] Department of Pharmacy, University of Genova, Viale Cembrano 4, 16148 Genova, Italy
[2] Department of Pharmacy, University of Salerno, Via Giovanni Paolo II 132, 84084 Salerno, Italy
[3] CERSAA Centro di Sperimentazione e Assistenza Agricola, Regione Rollo 98, 17031 Albenga, Italy
[4] Department of Informatics, Bioengineering, Robotics and System Science, University of Genova, Via Opera Pia 13, 16145 Genova, Italy

* Corresponding authors: Angela Bisio, e-mail address: angela.bisio@unige.it, Viale Cembrano 4, 16148 Genova, Italy; telephone number: +39 010 3352637;
Nunziatina De Tommasi, e-mail address: detommasi@unisa.it, Via Giovanni Paolo II 132, 84084 Salerno, Italy; telephone number: +39 089 969754.

1 Supplementary Figures and Tables

1.1 Supplementary Figures

Figure S1. Representative ^{1}H-NMR spectrum of Vessalico garlic.
Figure S2. Representative HSQC spectrum of Vessalico garlic.
Figure S3. Representative HMBC spectrum of Vessalico garlic.
Figure S4. Representative COSY spectrum of Vessalico garlic.
Figure S5. ^{1}H-NMR spectra of garlic accessions.
Figure S6. Results of PCA. CL and CCL variables.
Figure S7. Results of PCA applied to garlic accessions from different locations and farms.
Figure S8. Results of SOMs. Map clusterization for CL and CCL variables.
Figure S9. Results of SOMs. Graphical representation of map for CL and CCL variables.
Figure S10. Results of SOMs. U-matrix and maps for each CL and CCL variables.
Figure S11. Results of SOMs. Map clusterization for CL variables.
Figure S12. Results of SOMs. Graphical representation of map for CL variables.
Figure S13. Results of SOMs. U-matrix and maps for each CL variable.

1.2 Supplementary Tables

Table S1. List of garlic accessions used in the study.
Table S2. ^{1}H NMR chemical shifts (δ) and coupling constants (Hz) of Chenomx 600 MHz library (CL) and custom library (CCL) metabolites.

1.1 Supplementary Figures

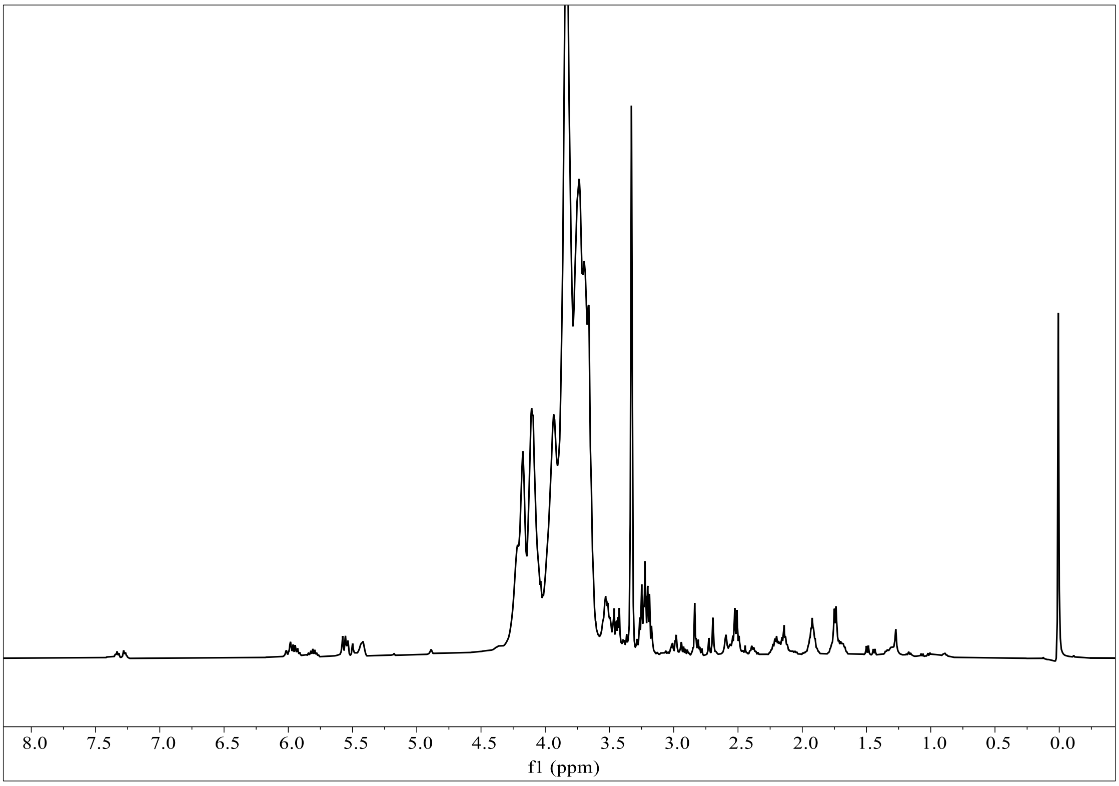

Figure S1. Representative ^{1}H-NMR spectrum of Vessalico garlic.
Accession 12 (Table S2) clove extract in CD_3OD-KH_2PO_4 in D_2O at pH 6.0, 600 MHz.

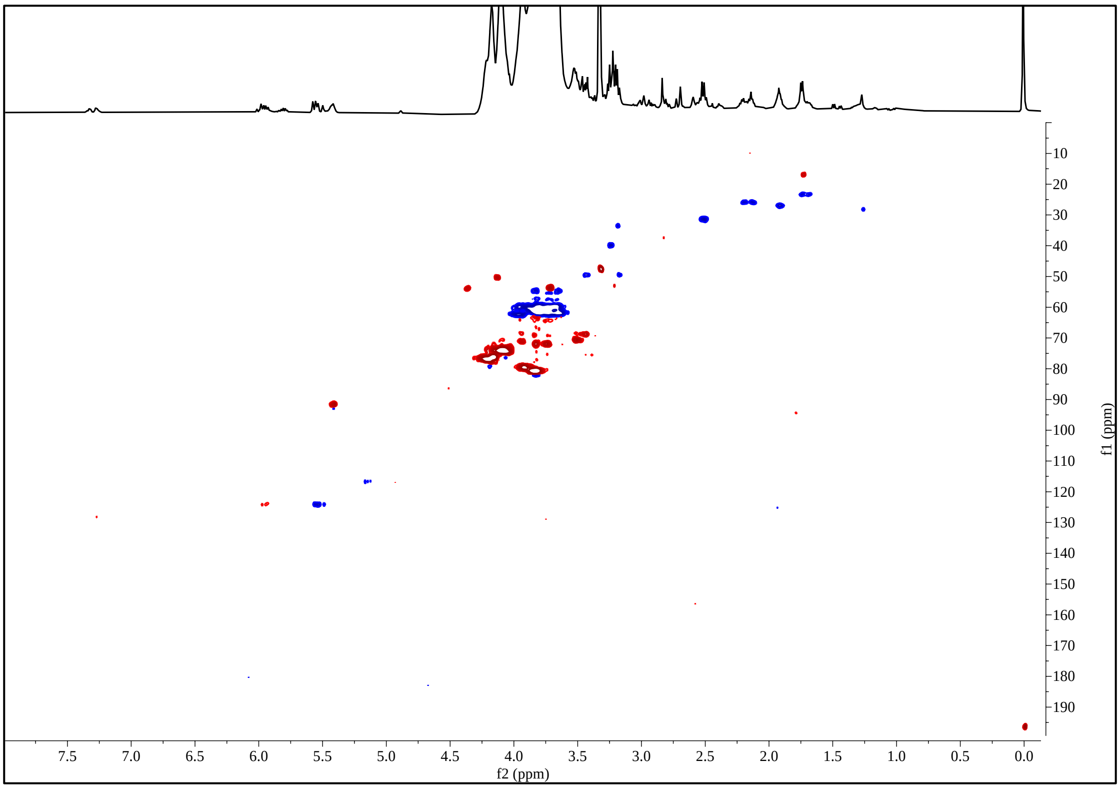

Figure S2. Representative HSQC spectrum of Vessalico garlic.
Accession 12 (Table S2) clove extract in CD_3OD-KH_2PO_4 in D_2O at pH 6.0, 600 MHz.

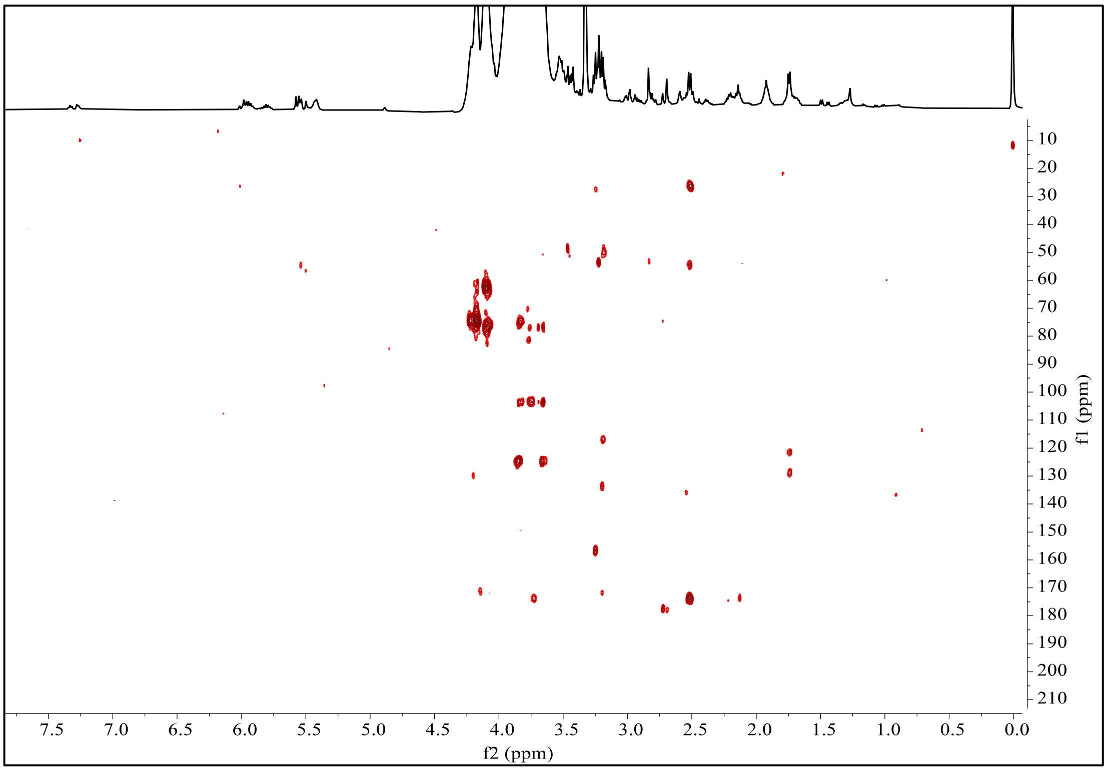

Figure S3. Representative HMBC spectrum of Vessalico garlic.
Accession 12 (Table S2) clove extract in CD_3OD-KH_2PO_4 in D_2O at pH 6.0, 600 MHz.

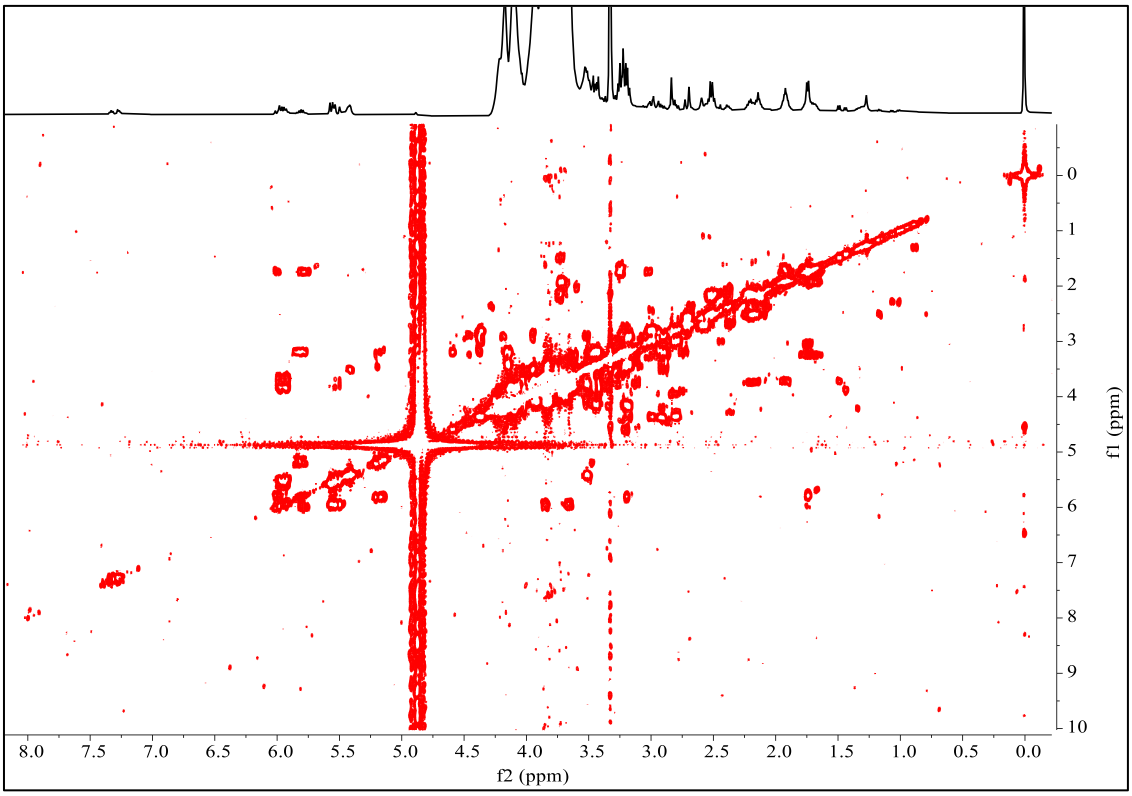

Figure S4. Representative COSY spectrum of Vessalico garlic.
Accession 12 (Table S2) clove extract in CD_3OD-KH_2PO_4 in D_2O at pH 6.0, 600 MHz.

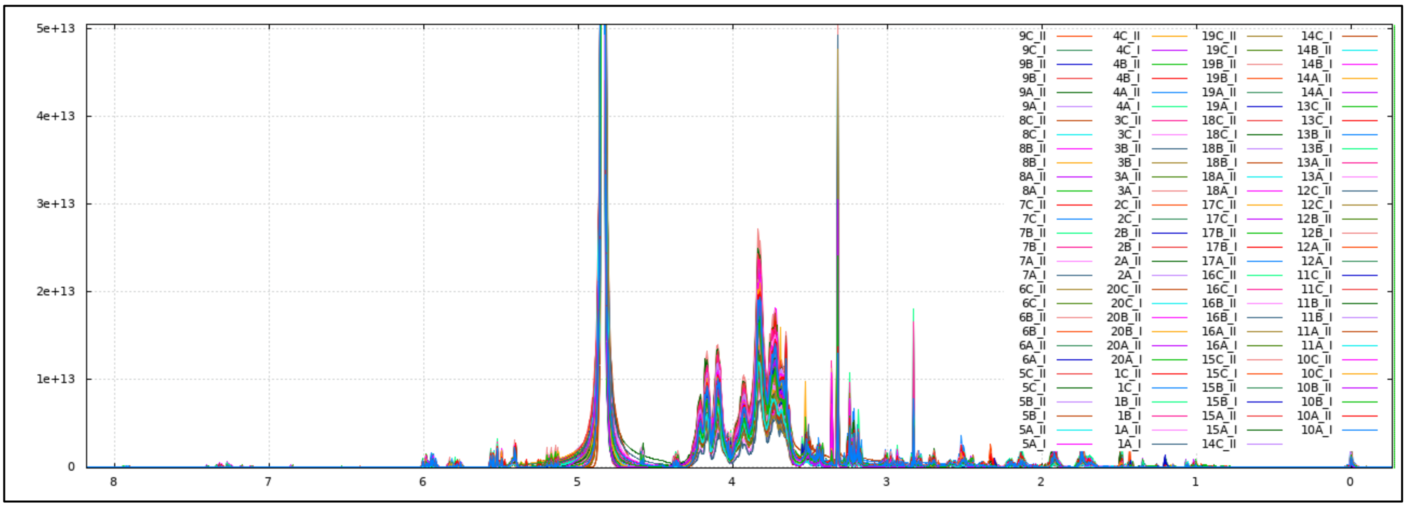

Figure S5. ^{1}H-NMR spectra of garlic accessions.
Spectra of the garlic extracts using the open access software NMRProcFlow v1.4.14. Spectra of the extracts are presented in different colours.

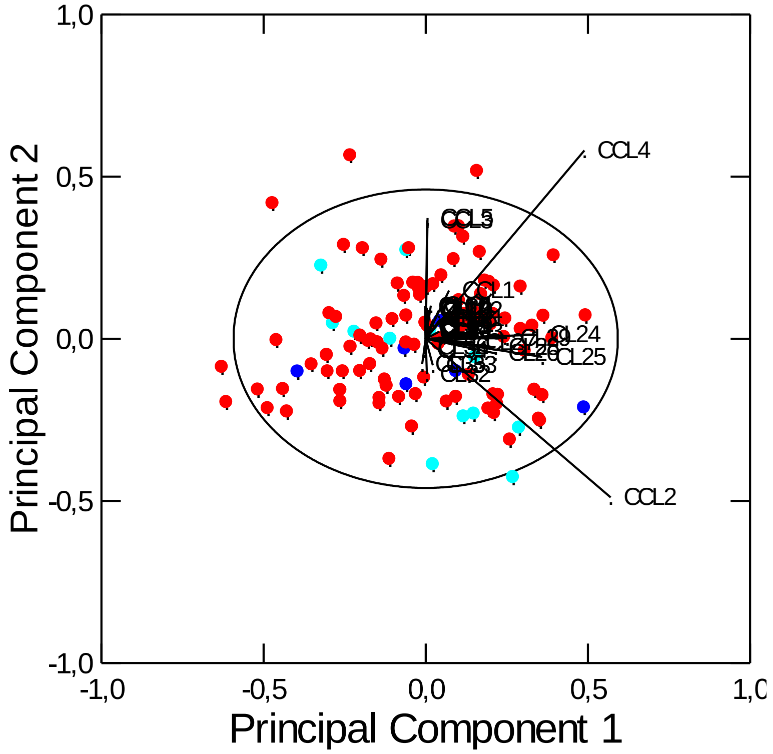

Figure S6. Results of PCA. CL and CCL variables.
The biplot shows the scores of the 120 spectra and the loadings of the 41 variables (Chenomx 600 MHz 36 library and 5 custom library metabolites) on Principal Components 1 and 2 (explaining 46.2% and 27.8% of the total variance, respectively): ● Vessalico; ● Messidor; ● Messidrôme). The ellipse represents the 95% confidence interval.

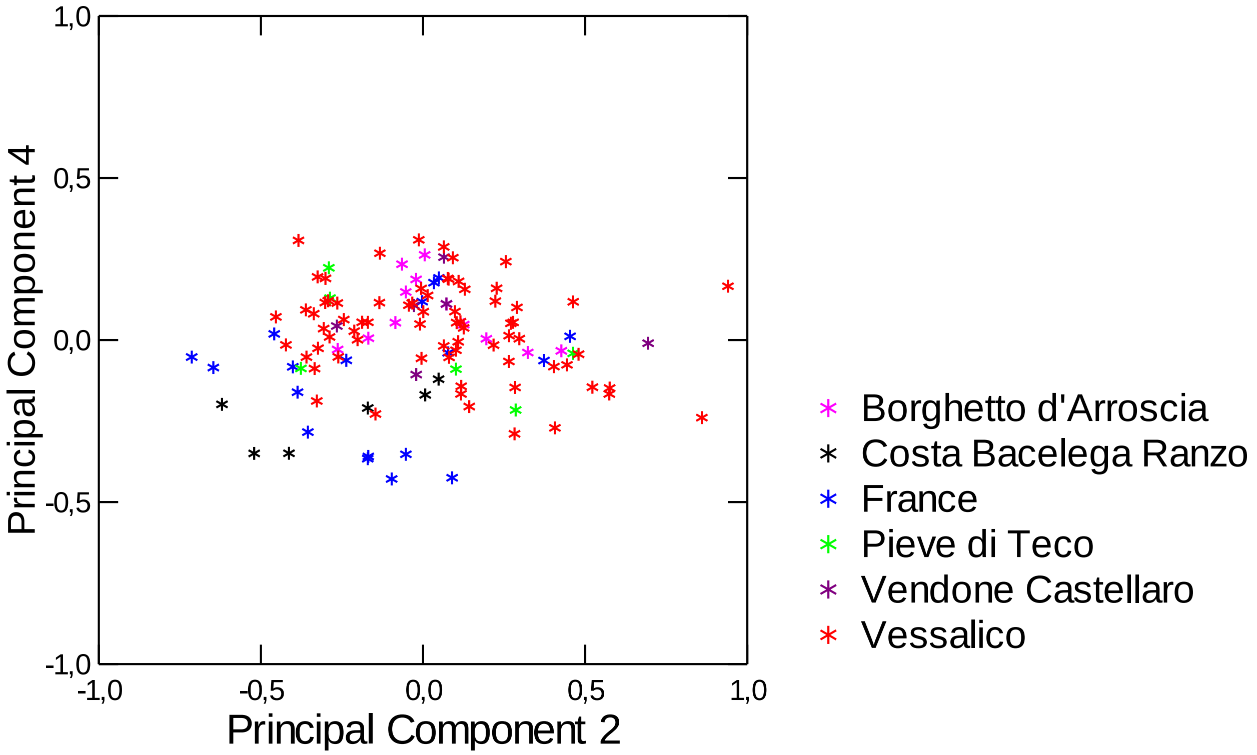

Figure S7. Results of PCA applied to garlic accessions from different locations and farms.
Plot of the scores of the samples described by Chenomx 600 MHz 36 library and 5 custom library metabolites (CL and CCL, Table S2) on Principal Components 2 and 4.

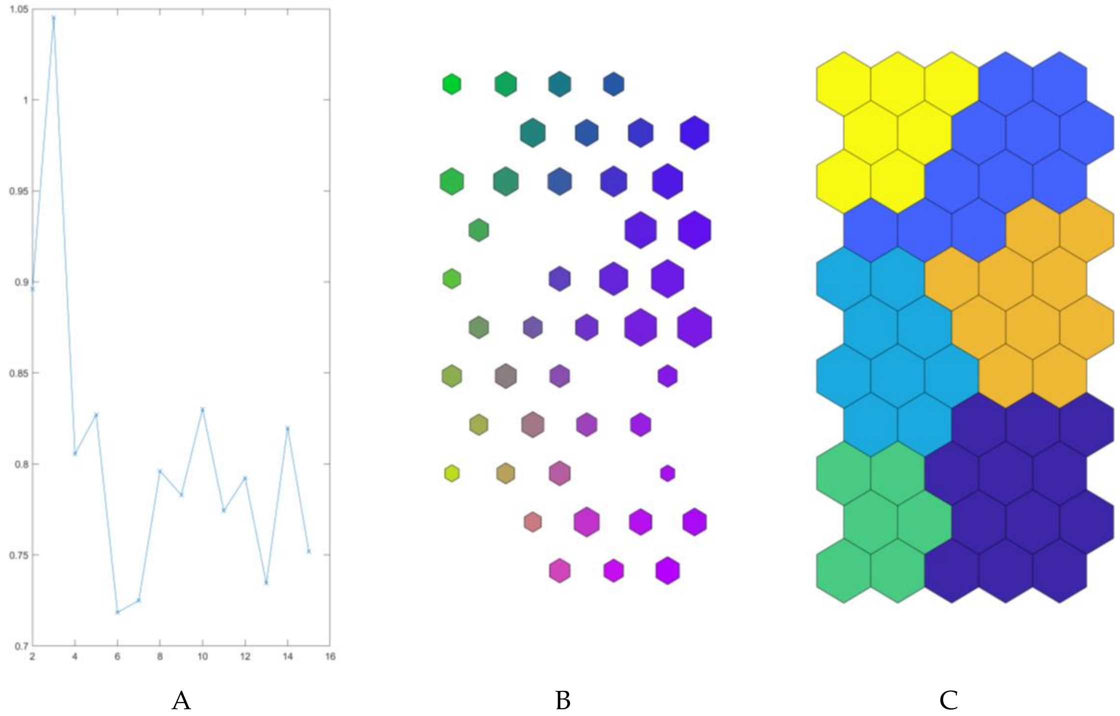

Figure S8. Results of SOMs. Map clusterization for CL and CCL variables.
Chenomx 600 MHz 36 library and 5 custom library metabolites (Table S2). A. Davies-Bouldine index progression: minimum value fits the best number of clusters. B. SOM output map with colour code association. Similar colours have similar characteristics. C. 6 clusters.

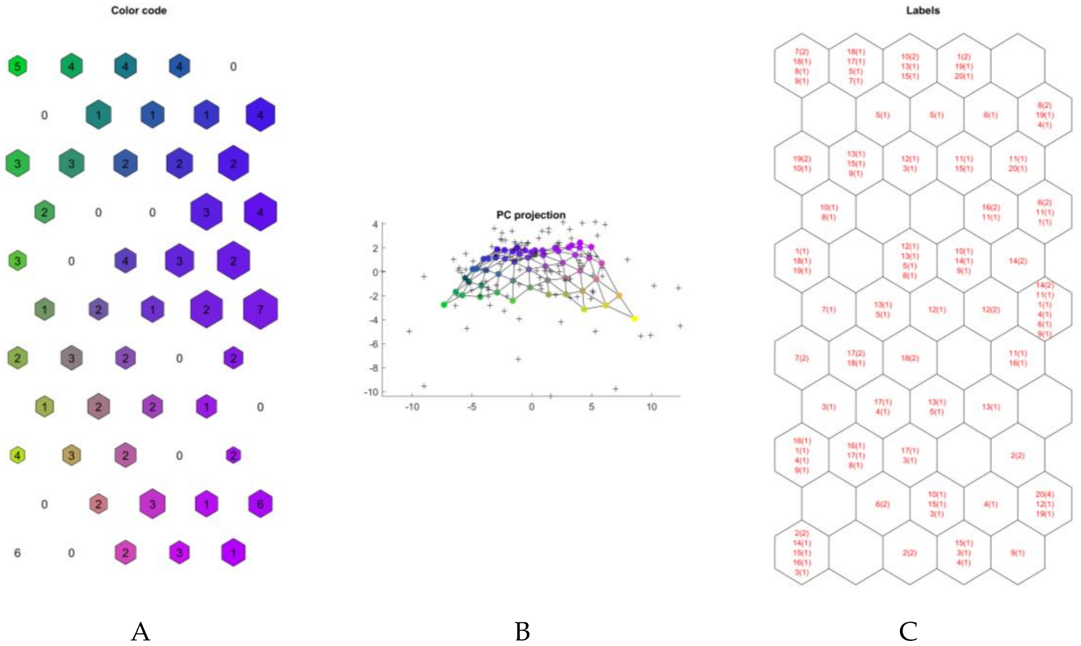

Figure S9. Results of SOMs. Graphical representation of map for CL and CCL variables.
Chenomx 600 MHz 36 library and 5 custom library metabolites (Table S2). A. SOM output map with colour code association. Similar colours have similar characteristics, numbers correspond to hit numbers. Dimensions of hexagons are related to the distance between neurons (biggest indicates greater distance); B. Principal Component projection of the map; C. labelled SOM output map, for each neuron the corresponding accession number (Table S1) and number of replicates (in parentheses) are shown.

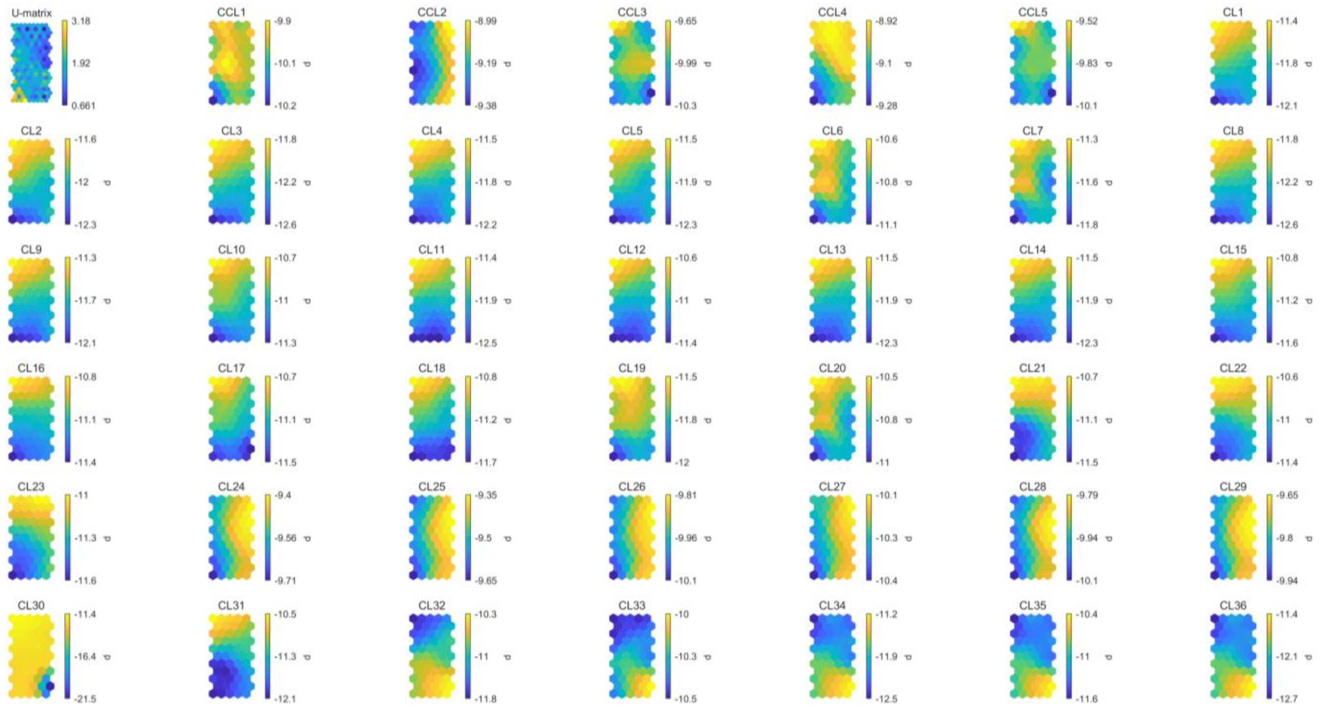

Figure S10. Results of SOMs. U-matrix and maps for each CL and CCL variables.
Chenomx 600 MHz 36 library and 5 custom library metabolites (Table S2). Similar color gradations indicate highly correlated variables.

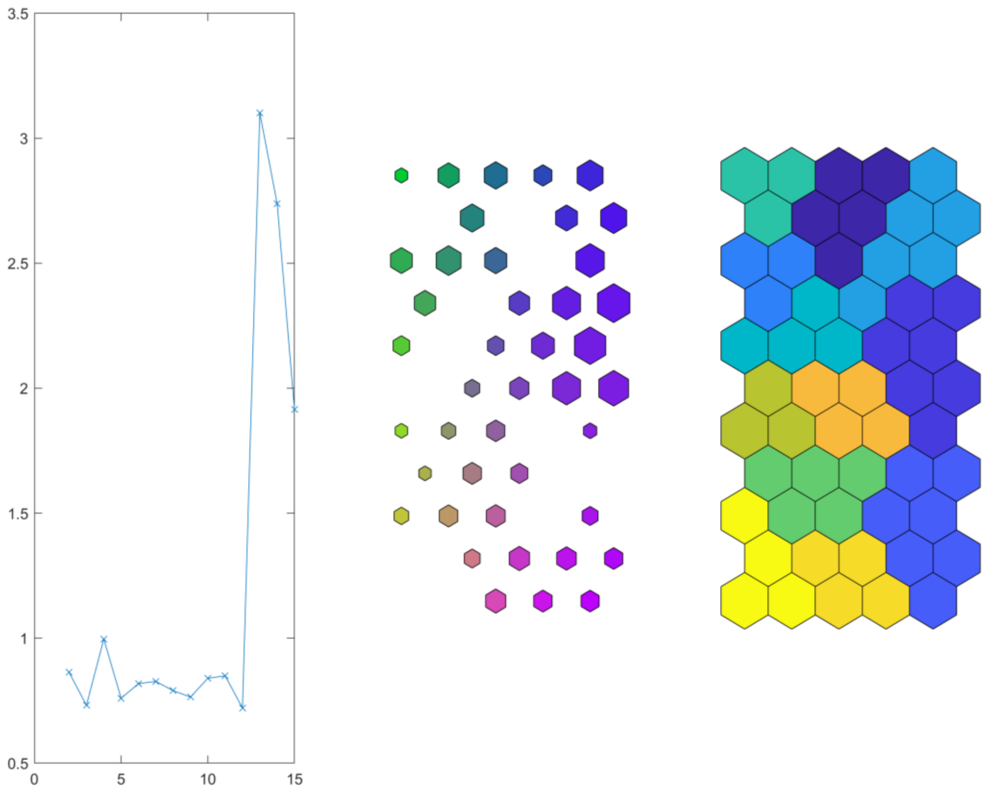

A B C

Figure S11. Results of SOMs. Map clusterization for CL variables.
Chenomx 600 MHz 36 library metabolites (Table S2). A. Davies-Bouldine index progression: minimum value fits the best number of clusters. B. SOM output map with colour code association. Similar colours have similar characteristics. C. 12 clusters.

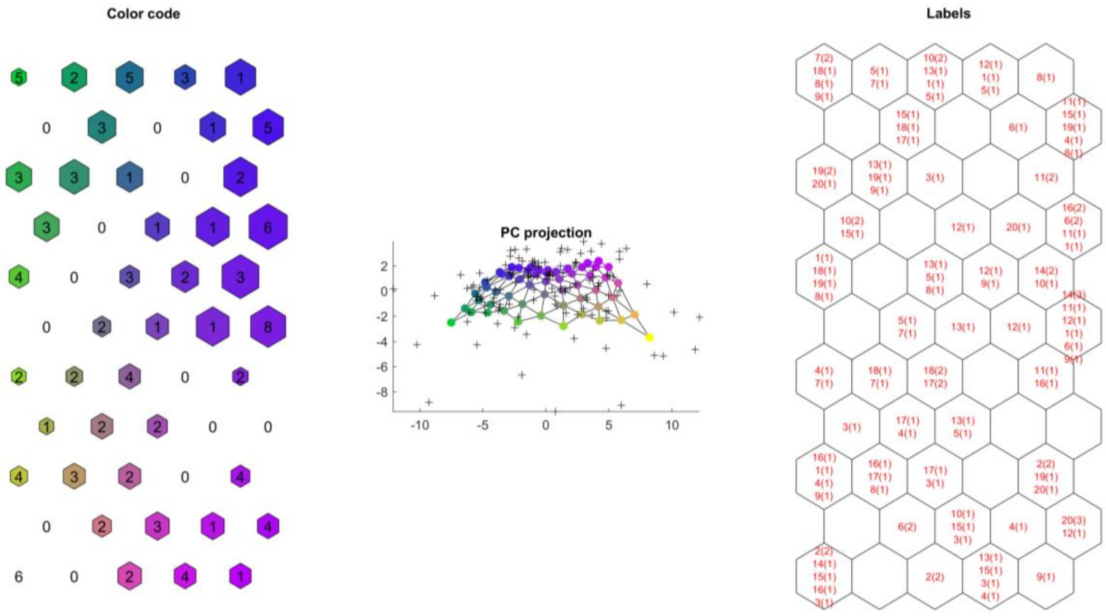

A B C

Figure S12. Results of SOMs. Graphical representation of map for CL variables.
Chenomx 600 MHz 36 library metabolites (Table S2). A. SOM output map with color code association. Similar colours have similar characteristics, numbers correspond to hit numbers. Dimensions of hexagons are related to the distance between neurons (biggest indicates greater distance); B. Principal Component projection of the map; C. labelled SOM output map, for each neuron the corresponding accession number (Table S1) and number of replicates (in parentheses) are shown.

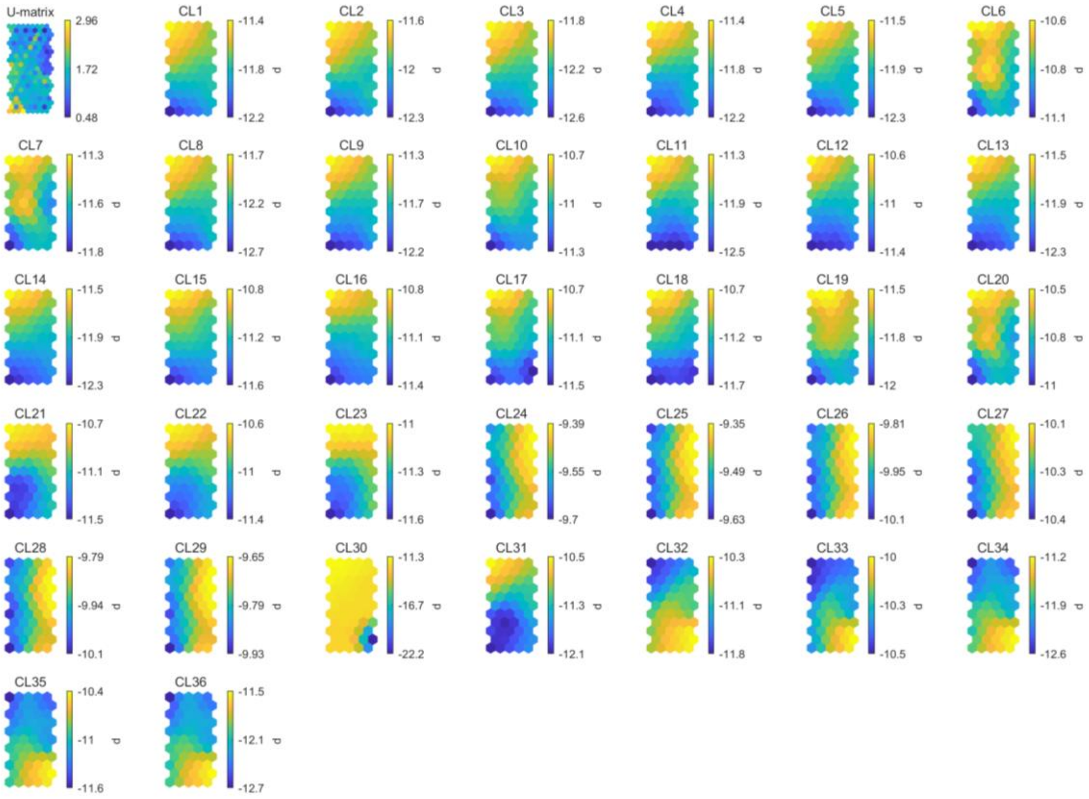

Figure S13. Results of SOMs. U-matrix and maps for each CL variable.
Chenomx 600 MHz 36library metabolites (Table S2). Similar color gradations indicate highly correlated variables.

1.2 Supplementary Tables

Table S1. List of garlic accessions used in the study.

Accession number	Ecotype or Cultivar name	Grower	Location	Breeding status (*)	Soil texture type	Yield (ton x ha^{-1})
1	Vessalico	A	Costa Bacelega Ranzo (Imperia, Italy) [a]	Ecotype	sandy clay loam soil	80 (**)
2	Vessalico	B	Vessalico (Imperia, Italy) [b]	Ecotype	sandy clay loam soil	80 (**)
3	Vessalico	C	Borghetto d'Arroscia (Imperia, Italy) [c]	Ecotype	sandy clay loam soil	80 (**)
4	Vessalico	D	Vessalico (Imperia, Italy) [b]	Ecotype	sandy clay loam soil	80 (**)
5	Vessalico	E	Vessalico (Imperia, Italy) [b]	Ecotype	sandy clay loam soil	80 (**)
6	Vessalico	F	Vessalico (Imperia, Italy) [b]	Ecotype	sandy clay loam soil	80 (**)
7	Vessalico	G	Vessalico (Imperia, Italy) [b]	Ecotype	sandy clay loam soil	80 (**)
8	Vessalico	H	Vendone fraz. Castellaro (Imperia, Italy)	Ecotype	sandy clay loam soil	80 (**)
9	Vessalico	I	Vessalico (Imperia, Italy) [b]	Ecotype	sandy clay loam soil	80 (**)
10	Vessalico	J	Vessalico (Imperia, Italy) [b]	Ecotype	sandy clay loam soil	80 (**)
11	Vessalico	K	Vessalico (Imperia, Italy) [b]	Ecotype	sandy clay loam soil	80 (**)
12	Vessalico	L	Vessalico (Imperia, Italy) [b]	Ecotype	sandy clay loam soil	80 (**)
13	Vessalico	M	Vessalico (Imperia, Italy) [b]	Ecotype	sandy clay loam soil	80 (**)
14	Vessalico	N	Borghetto d'Arroscia (Imperia, Italy) [c]	Ecotype	sandy clay loam soil	80 (**)
15	Vessalico	O	Pieve di Teco (Imperia, Italy) [d]	Ecotype	sandy clay loam soil	80 (**)
16	Vessalico	P	Vessalico (Imperia, Italy) [b]	Ecotype	sandy clay loam soil	80 (**)
17	Vessalico	Q	Vessalico (Imperia, Italy) [b]	Ecotype	sandy clay loam soil	80 (**)
18	Messidor	R	France	Released variety	-	9-12 (***)
19	Messidrôme	S	France	Released variety	-	8-14 (***)
20	Messidor	T	France	Released variety	-	9-12 (***)

[a] Coordinates of the downtown of the village: 44°04′51′′N; 8°01′51′′E; elevation 450 (m.a.s.l).
[b] Coordinates of the downtown of the village: 44°02′46′′N; 7°57′43′′E; elevation 197 (m.a.s.l).
[c] Coordinates of the downtown of the village: 44°03′26′′N; 7°58′54′′E; elevation 155 (m.a.s.l).
[d] Coordinates of the downtown of the village: 44°02′52′′N; 7°54′57′′E; elevation 240 (m.a.s.l).
* Landrace / Farmers' variety / Breeding line / Released variety/ ecotype.
** Maximum production capacity; only the cultivated area (excluding passages and other cultivation tares); included the fresh aerial part of the plant (foliage).
*** From breeder catalogue (INRA). Excluded aerial part of the plant; included passages and cultivation tares.

Cultivars	Foliage Colour **	Foliage Attitude ***	Bulb skin color ****	Bulb (cm)	Bulb Weight(g)	Shape	Colour of tunic	No. of bulblets	Clove size (cm)	Clove Weight (g)	Garlic flavor (+)
Vessalico*	Dark green	Erect	White	3,5-8	50-100	Oval	White/cream	10-15	3-5	5-10	+
Messidor°	Dark green	Erect	White	8-10	40-60	Oval	White/cream	10-15	2-3	2-5	++
Messidrôme°	Dark green	Erect	White	12-15	>60	Oval	White/cream	10-15	2-3	3-5	++

* From PGI production specification.
° From breeder catalogue (INRA).
** Dark green / Light green.
*** Erect / Droop.
****Purple tinge / White.

The best climatic conditions for garlic cultivation in Liguria are found in the inland areas, away from the sea, but characterized by a Mediterranean climate: a limited day-night temperature range between 5 and 10 °C; relative humidity of the air between 50 and 75%; rainfall concentrated mainly in the spring period (February-April), before maturation, and in autumn (September-October), before bulb planting.
The soil, from a chemical-physical point of view, is characteristic of the maritime slopes of the Ligurian Apennines and Alps: calcareous, rich in skeletal material, with a high clay and sandy component, with a modest water supply, especially in the period approaching harvest.
All these conditions, combined with the adoption of cultivation techniques refined over time by generations of farmers, have converged in a mountainous area behind Imperia (Italy) and identifiable with the territory of Vessalico (IM) and other 10 municipalities of the Arroscia Valley (IM): Vessalico, Aquila d'Arroscia, Armo, Borghetto d'Arroscia, Cosio d'Arroscia, Mendatica, Montegrosso Pian Latte, Pornassio, Pieve di Teco, Ranzo, Rezzo.
The garlic cultivated in that area has always been recognized under the name of the municipality of Vessalico which, due to its strategic position at the crossroads of ancient trade routes, has always been the center of trade for this product.
The cultivation technique of Vessalico garlic is detailed in the Vessalico Garlic Specification for Protected Geographical Indication (PGI) candidacy. Below is a summary.
Vessalico garlic is cultivated in a hilly environment characterized by small, terraced plots, typical of the Ligurian hinterland. The soil has a medium texture and a good presence of skeletal material. Crop rotation is essential to avoid phytosanitary problems, and it is recommended to alternate garlic with other crops for at least two cycles. Vessalico garlic can be cultivated using both conventional and organic or biodynamic methods, complying with current regulations. Soil preparation occurs in the summer through plowing and refinement, with the possible addition of organic matter. Planting is preferably done in autumn, by February, and can be done manually or with specific machinery. It is important to follow the indications for planting depth and spacing. After planting, one or two spring hoeing operations are necessary to eliminate weeds, preferably with eco-sustainable techniques. Emergency irrigation is allowed in case of drought, but it is advisable to limit water supply before harvesting to promote bulb maturation. Fertilization can be done with mature organic substances or mineral fertilizers, depending on crop needs. Harvesting takes place when garlic leaves begin to yellow and dry, and it can be done manually or mechanically. Plants are then selected based on size and subjected to a slow drying period to preserve quality. Vessalico garlic is then handcrafted into characteristic "reste" (braids) and stored in cool, dry environments until commercialization.

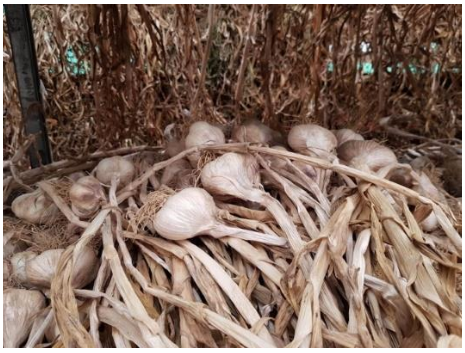

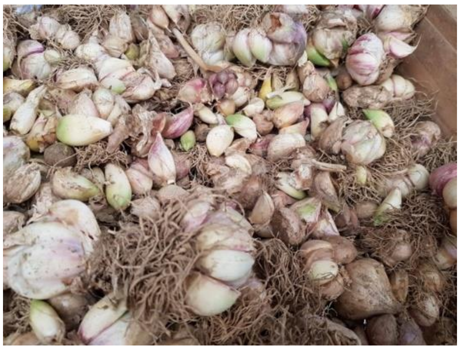

Table S2. ^{1}H NMR chemical shifts (δ) and coupling constants (Hz) of Chenomx 600 MHz library (CL) and custom library (CCL) metabolites.
NMR spectra of CCL metabolites were recorded in CD_3OD-KH_2PO_4 in D_2O at pH 6.0, 600 MHz.

CCL/CL number	metabolite	selected characteristic NMR chemical shifts in ppm - cluster midpoint (multiplicity, *J*)	other chemical shifts in ppm - cluster midpoint (multiplicity, *J*)	MSI Status [b]	references
**CCL1**	*S*-methyl-L-cysteine	2.17 (3H, s)	2.97 [a], 3.86 [a]	1	(Higuchi et al., 2003)
**CCL2**	methiin	2.83 (3H, s)	3.25 [a], 3.48 [a], 4.13 [a]	1	(Hibi et al., 2013; Ingallina et al., 2023)
**CCL3**	*S*-allyl-L-cysteine	5.17 (1H) [a]	2.92 [a], 3.10 [a], 3.23 [a], 3.77 [a], 5.24 [a], 5.83 [a]	1	(Higuchi et al., 2003; Maldonado et al., 2011)
**CCL4**	L-alliin	5.50 (1H) [a]	3.21 [a], 3.66 [a], 3.82 [a], 4.09 [a], 5.53 [a], 5.96 [a]	1	(Hibi et al., 2013)
**CCL5**	allicin	5.88 (1H) [a]	3.13 [a], 3.32 [a], 3.37 [a], 3.60 [a], 5.28 [a]	1	(Pacholczyk-Sienicka et al., 2024; Ritota et al., 2012)
**CL1**	leucine	0.95 (3H) [a]	0.94 [a],1.67 [a], 1.71 [a], 1.73 [a], 3.73 [a]	2	(Le Mao et al., 2021; Liang et al., 2015; Pacholczyk-Sienicka et al., 2024; Verpoorte et al., 2007)
**CL2**	isoleucine	1.00 (3H, d, *J* = 7.0)	0.93 [a], 1.25 [a], 1.46 [a], 1.97 [a], 3.66 [a]	2	(Le Mao et al., 2021; Liang et al., 2015; Pacholczyk-Sienicka et al., 2024)
**CL3**	valine	1.03 (3H, d, *J* = 7.0)	0.98 [a], 2.26 [a], 3.60 [a]	2	(Liang et al., 2015; Pacholczyk-Sienicka et al., 2024; Verpoorte et al., 2007)
**CL4**	threonine	1.32 (3H, d, *J* = 6.6)	3.58 [a], 4.25 [a]	2	(Liang et al., 2015; Pacholczyk-Sienicka et al., 2024; Verpoorte et al., 2007)
**CL6**	alanine	1.47 (3H, d, *J* = 7.2)	3.77 [a]	2	(Liang et al., 2015; Pacholczyk-Sienicka et al., 2024)
**CL5**	lysine	1.71 (2H) [a]	1.43 [a], 1.50 [a], 1.88 [a], 1.91 [a], 3.02 [a], 3.75 [a]	2	(Liang et al., 2015; Pacholczyk-Sienicka et al., 2024)
**CL7**	acetic acid	1.91 (3H) [a]		2	(Fulmer et al., 2010)
**CL8**	acetamide	1.99 (3H) [a]		2	(Matsuoka et al., 2015)
**CL9**	homoserine	2.01 (2H) [a]	2.14 [a], 3.77 [a], 3.85 [a]	2	(Jamieson et al., 2009; Wang et al., 2008)
**CL10**	glutamine	2.11 (1H) [a]	2.14 [a], 2.43 [a], 2.46 [a], 3.77 [a]	2	(Liang et al., 2015; Pacholczyk-Sienicka et al., 2024; Verpoorte et al., 2007)
**CL11**	succinylacetone	2.26 (3H) [a]	2.41 [a], 2.81 [a], 3.85 [a]	2	(Bal et al., 2009)
**CL12**	glutamic acid	2.33 (1H) [a]	2.04 [a], 2.12 [a], 2.36 [a], 3.75 [a]	2	(Liang et al., 2015; Lucas-Torres et al., 2018; Sabino et al., 2019; Verpoorte et al., 2007)
**CL13**	pyruvic acid	2.36 (3H) [a]		2	(Bourafai-Aziez et al., 2022; Le Mao et al., 2021)
**CL14**	succinic acid	2.39 (4H) [a]		2	(Gaviglio and Doctorovich, 2008; Kostidis et al., 2017)
**CL15**	riboflavin	2.48 (3H) [a]	2.58 [a], 3.73 [a], 3.87 [a], 3.93 [a], 3.97 [a], 4.44 [a], 4.95 [a], 5.14 [a], 7.96 [a], 7.98 [a]	2	(Jiménez-Nava et al., 2023)
**CL16**	citric acid	2.69 (2H) [a]	2.53 [a]	2	(Le Mao et al., 2021; Pacholczyk-Sienicka et al., 2024; Verpoorte et al., 2007)
**CL17**	aspartic acid	2.80 (1H) [a]	2.67 [a], 3.89 [a]	2	(Liang et al., 2015; Pacholczyk-Sienicka et al., 2024)
**CL18**	asparagine	2.94 (1H) [a]	2.85 [a], 4.00 [a]	2	(Liang et al., 2015; Lucas-Torres et al., 2018; Pacholczyk-Sienicka et al., 2024)
**CL19**	choline	3.19 (9H, s)	3.51 [a], 4.06 [a]	2	(Le Mao et al., 2021; Liang et al., 2015)
**CL20**	arginine	3.24 (2H) [a]	1.64 [a], 1.72 [a], 1.89 [a], 1.92 [a], 3.76 [a]	2	(Nilsson et al., 2004)
**CL21**	cystine	3.38 (2H) [a]	3.18 [a], 4.11 [a]	2	(Lucas-Torres et al., 2018)
**CL22**	proline	3.41 (1H) [a]	1.98 [a], 2.02 [a], 2.06 [a], 2.34 [a], 3.33 [a], 4.12 [a]	2	(Le Mao et al., 2021; Liang et al., 2015; Verpoorte et al., 2007)
**CL23**	glycine	3.55 (2H, s)		2	(Kostidis et al., 2017)

**CL24**	gluconic acid	3.77 (1H) [a]	3.66 [a], 3.75 [a], 3.82 [a], 4.03 [a], 4.13 [a]	2	(Horton et al., 1983; Ramos et al., 2000)
**CL25**	methionine	3.85(1H) [a]	2.11 [a], 2.13 [a], 2.19 [a], 2.63 [a]	2	(Kostidis et al., 2017)
**CL26**	serine	3.94 (1H)	3.84 [a], 3.98 [a]	2	(Kostidis et al., 2017)
**CL27**	fructose	4.01(2H) [a]	3.56 [a], 3.70 [a], 3.78 [a], 3.88 [a], 3.99 [a], 4.09 [a]	2	(Le Mao et al., 2021)
**CL28**	lactic acid	4.11 (1H) [a]	1.32 (3H) [a]	2	(Le Mao et al., 2021; Liang et al., 2015)
**CL29**	pyroglutamic acid	4.17 (1H) [a]	2.02 [a], 2.38 [a], 2.41 [a], 2.50 [a]	2	(Kostidis et al., 2017; Liang et al., 2015)
**CL30**	trigonelline	4.43 (3H, s)	8.07 [a], 8.82 [a], 8.83 [a], 9.11 [a]	2	(Le Mao et al., 2021; Ritota et al., 2012; Sabino et al., 2019)
**CL31**	xylose	4.57 (1H, d, *J* = 3.75)	3.22 [a], 3.31 [a], 3.43 [a], 3.52 (1H) [a], 3.60 [a], 3.62 [a], 3.66 [a], 3.67 [a], 3.69 [a], 3.92 [a], 5.19 (1H, d, *J* = 3.66)	2	(Le Mao et al., 2021; Moing et al., 2004)
**CL32**	α-glucose	5.23 (1H, d, *J* = 3.75)	3.23 [a], 3.39 [a], 3.45 [a], 3.48 [a], 3.52 [a], 3.70 [a], 3.72 [a], 3.82 [a], 3.89 [a], 4.64 [a]	2	(Bourafai-Aziez et al., 2022; Le Mao et al., 2021)
**CL33**	sucrose	5.40 (1H, d, *J* = 3.85)	3.47 [a], 3.55 [a], 3.67 [a], 3.76 [a], 3.80 [a], 3.82 [a], 3.83 [a], 3.88 (1H) [a], 4.04 [a], 4.21 [a]	2	(Sabino et al., 2019)
**CL34**	fumaric acid	6.51 (2H, s)		2	(Pacholczyk-Sienicka et al., 2024)
**CL35**	histidine	7.09 (1H, s)	3.14 [a], 3.24 [a], 3.98 [a], 7.87 [a]	2	(Pacholczyk-Sienicka et al., 2024)
**CL36**	phenylalanine	7.42 (2H, m)	3.12 [a], 3.28 [a], 3.99 [a], 7.32 [a], 7.37 [a]	2	(Kostidis et al., 2017)

[a] Overlapped signals
[b] MSI level of identification according to Sumner *et al*. (Sumner et al., 2007).

References

Bal, D., Kraska-Dziadecka, A., Gryff-Keller, A., 2009. Solution structure of succinylacetone, an unsymmetrical beta-diketone, as studied by 13C NMR and GIAO-DFT calculations. J. Org. Chem. 74(22), 8604-8609.

Bourafai-Aziez, A., Jacob, D., Charpentier, G., Cassin, E., Rousselot, G., Moing, A., Deborde, C., 2022. Development, validation, and use of 1H-NMR spectroscopy for evaluating the quality of acerola-based food supplements and quantifying ascorbic acid. Molecules 27(17), 5614.

Fulmer, G.R., Miller, A.J.M., Sherden, N.H., Gottlieb, H.E., Nudelman, A., Stoltz, B.M., Bercaw, J.E., Goldberg, K.I., 2010. NMR chemical shifts of trace impurities: Common laboratory solvents, organics, and gases in deuterated solvents relevant to the organometallic chemist. Organometallics 29(9), 2176-2179.

Gaviglio, C., Doctorovich, F., 2008. Hydrogen-free homogeneous catalytic reduction of olefins in aqueous solutions. The Journal of Organic Chemistry 73(14), 5379-5384.

Hibi, M., Kawashima, T., Yajima, H., Smirnov, S.V., Kodera, T., Sugiyama, M., Shimizu, S., Yokozeki, K., Ogawa, J., 2013. Enzymatic synthesis of chiral amino acid sulfoxides by Fe(II)/α-ketoglutarate-dependent dioxygenase. Tetrahedron: Asymmetry 24(17), 990-994.

Higuchi, O., Tateshita, K., Nishimura, H., 2003. Antioxidative activity of sulfur-containing compounds in *Allium* species for human low-density lipoprotein (ldl) oxidation *in vitro*. J. Agric. Food. Chem. 51(24), 7208-7214.

Horton, D., Wałaszek, Z., Ekiel, I., 1983. Conformations of D-gluconic, D-mannonic, and D-galactonic acids in solution, as determined by N.M.R. spectroscopy. Carbohydr. Res. 119, 263-268.

Ingallina, C., Di Matteo, G., Spano, M., Acciaro, E., Campiglia, E., Mannina, L., Sobolev, A.P., 2023. Byproducts of globe artichoke and cauliflower production as a new source of bioactive compounds in the green economy perspective: an NMR study. Molecules 28(3), 1363.

Jamieson, A.G., Boutard, N., Beauregard, K., Bodas, M.S., Ong, H., Quiniou, C., Chemtob, S., Lubell, W.D., 2009. Positional scanning for peptide secondary structure by systematic solid-phase synthesis of amino lactam peptides. Journal of the American Chemical Society 131(22), 7917-7927.

Jiménez-Nava, R.A., Zepeda-Vallejo, L.G., Santoyo-Tepole, F., Chávez-Camarillo, G.M., Cristiani-Urbina, E., 2023. RP-HPLC separation and 1H NMR identification of a yellow fluorescent compound - Riboflavin (vitamin B2) produced by the yeast *Hyphopichia wangnamkhiaoensis*. Biomolecules 13(9), 1423.

Kostidis, S., Addie, R.D., Morreau, H., Mayboroda, O.A., Giera, M., 2017. Quantitative NMR analysis of intra- and extracellular metabolism of mammalian cells: A tutorial. Anal. Chim. Acta 980, 1-24.

Le Mao, I., Martin-Pernier, J., Bautista, C., Lacampagne, S., Richard, T., Da Costa, G., 2021. 1H-NMR Metabolomics as a tool for winemaking monitoring. Molecules 26(22), 6771.

Liang, T., Wei, F., Lu, Y., Kodani, Y., Nakada, M., Miyakawa, T., Tanokura, M., 2015. Comprehensive NMR Analysis of Compositional Changes of Black Garlic during Thermal Processing. J. Agric. Food. Chem. 63(2), 683-691.

Lucas-Torres, C., Huber, G., Ichikawa, A., Nishiyama, Y., Wong, A., 2018. HR-μMAS NMR-Based Metabolomics: Localized Metabolic Profiling of a Garlic Clove with μg Tissues. Anal. Chem. 90(22), 13736-13743.

Maldonado, P.D., Alvarez-Idaboy, J.R., Aguilar-González, A., Lira-Rocha, A., Jung-Cook, H., Medina-Campos, O.N., Pedraza-Chaverrí, J., Galano, A., 2011. Role of Allyl group in the hydroxyl and peroxyl radical scavenging activity of s-allylcysteine. The Journal of Physical Chemistry B 115(45), 13408-13417.

Matsuoka, A., Isogawa, T., Morioka, Y., Knappett, B.R., Wheatley, A.E.H., Saito, S., Naka, H., 2015. Hydration of nitriles to amides by a chitin-supported ruthenium catalyst. RSC Advances 5(16), 12152-12160.

Moing, A., Maucourt, M., Renaud, C., Gaudill, egrave, re, M., Brouquisse, R., Lebouteiller, B., Gousset-Dupont, A., Vidal, J., Granot, D., Denoyes-Rothan, B., Lerceteau-K, ouml, hler, E., Rolin, D., 2004. Quantitative metabolic profiling by 1-dimensional 1H-NMR analyses: application to plant genetics and functional genomics. Funct. Plant Biol. 31(9), 889-902.

Nilsson, M., Duarte, I.F., Almeida, C., Delgadillo, I., Goodfellow, B.J., Gil, A.M., Morris, G.A., 2004. High-resolution NMR and diffusion-ordered spectroscopy of port wine. J. Agric. Food. Chem. 52(12), 3736-3743.

Pacholczyk-Sienicka, B., Modranka, J., Ciepielowski, G., 2024. Comparative analysis of bioactive compounds in garlic owing to the cultivar and origin. Food Chem. 439, 138141.

Ramos, L.M., Caldeira, M.M., Gil, V.M., 2000. NMR study of the complexation of D-gulonic acid with tungsten(VI) and molybdenum(VI). Carbohydr. Res. 329(2), 387-397.

Ritota, M., Casciani, L., Han, B.-Z., Cozzolino, S., Leita, L., Sequi, P., Valentini, M., 2012. Traceability of Italian garlic (Allium sativum L.) by means of HRMAS-NMR spectroscopy and multivariate data analysis. Food Chem. 135(2), 684-693.

Sabino, A.R., Tavares, S., Riffel, A., Li, J.V., Oliveira, D.J., Feres, C., Henrique, L., Oliveira, J.S., Correia, G.D.S., Sabino, A.S., Nascimento, T.G., Hawkes, G., Santana, A.E.G., Holmes, E., Bento, E.S., 2019. Short communication 1 H NMR metabolomic approach reveals chlorogenic acid as a response of sugarcane induced by exposure to Diatraea saccharalis. Ind. Crop. Prod. 140, 1-6.

Sumner, L.W., Amberg, A., Barrett, D., Beale, M.H., Beger, R., Daykin, C.A., Fan, T.W., Fiehn, O., Goodacre, R., Griffin, J.L., Hankemeier, T., Hardy, N., Harnly, J., Higashi, R., Kopka, J., Lane, A.N., Lindon, J.C., Marriott, P., Nicholls, A.W., Reily, M.D., Thaden, J.J., Viant, M.R., 2007. Proposed minimum reporting standards for chemical analysis Chemical Analysis Working Group (CAWG) Metabolomics Standards Initiative (MSI). Metabolomics 3(3), 211-221.

Verpoorte, R., Choi, Y.H., Kim, H.K., 2007. NMR-based metabolomics at work in phytochemistry. Phytochemistry Reviews 6(1), 3-14.

Wang, M.-C., Zhang, Q.-J., Zhao, W.-X., Wang, X.-D., Ding, X., Jing, T.-T., Song, M.-P., 2008. Evaluation of enantiopure N-(ferrocenylmethyl)azetidin-2-yl(diphenyl)methanol for catalytic asymmetric addition of organozinc reagents to aldehydes. The Journal of Organic Chemistry 73(1), 168-176.
